# 1-(4-Chloro­phen­yl)-2-[(3-phenyl­isoquinolin-1-yl)sulfan­yl]ethanone

**DOI:** 10.1107/S1600536809000257

**Published:** 2009-01-08

**Authors:** P. Manivel, Venkatesha R. Hathwar, P. Nithya, R. Subashini, F. Nawaz Khan

**Affiliations:** a Chemistry Division, School of Science and Humanities, VIT University, Vellore 632 014, Tamil Nadu, India; b Solid State and Structural Chemistry Unit, Indian Institute of Science, Bangalore 560 012, Karnataka, India

## Abstract

The title compound, C_23_H_16_ClNOS, exhibits dihedral angles of 11.73 (1) and 66.07 (1)°, respectively, between the mean plane of the isoquinoline system and the attached phenyl ring, and between the isoquinoline system and the chloro­phenyl ring. The dihedral angle between the phenyl and chlorophenyl rings is 54.66 (1)°.

## Related literature

For general background, see: Cremlyn *et al.* (1996 ▶); Carreno (1995 ▶); Kondo *et al.* (2000 ▶); Mosberg & Omnaas (1985 ▶); McReynolds *et al.* (2004 ▶). For related crystal structures, see: Hathwar *et al.* (2008 ▶); Manivel *et al.* (2009 ▶).
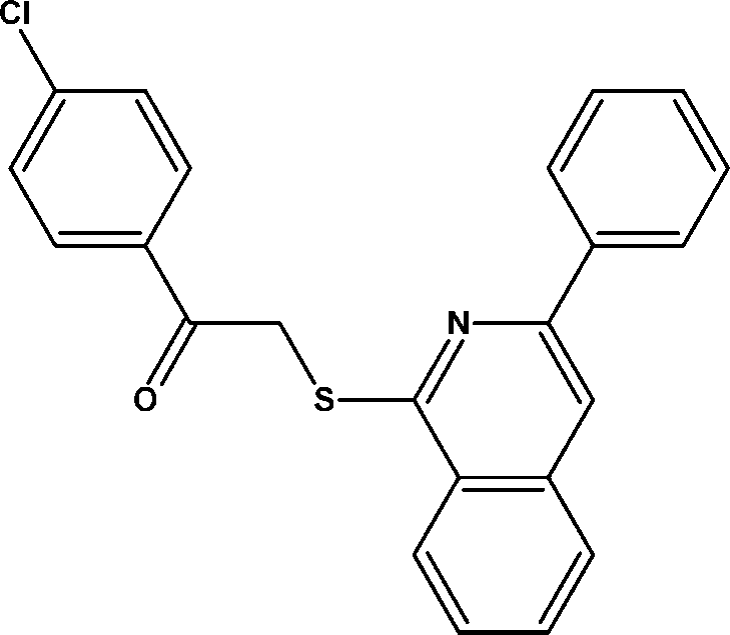


## Experimental

### 

#### Crystal data

C_23_H_16_ClNOS
*M* *_r_* = 389.89
Orthorhombic, 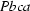

*a* = 9.5874 (14) Å
*b* = 17.888 (3) Å
*c* = 22.025 (3) Å
*V* = 3777.3 (10) Å^3^
*Z* = 8
Mo *K*α radiation
μ = 0.33 mm^−1^
*T* = 290 (2) K
0.26 × 0.20 × 0.14 mm

#### Data collection

Bruker SMART CCD area-detector diffractometer
Absorption correction: multi-scan (*SADABS*; Sheldrick, 1996 ▶) *T*_min_ = 0.901, *T*_max_ = 0.956
25674 measured reflections
3332 independent reflections
1915 reflections with *I* > 2σ(*I*)
*R*_int_ = 0.116

#### Refinement

*R*[*F* ^2^ > 2σ(*F* ^2^)] = 0.071
*wR*(*F* ^2^) = 0.125
*S* = 1.04
3332 reflections
244 parameters
H-atom parameters constrained
Δρ_max_ = 0.23 e Å^−3^
Δρ_min_ = −0.23 e Å^−3^

### 

Data collection: *SMART* (Bruker, 2004 ▶); cell refinement: *SAINT* (Bruker, 2004 ▶); data reduction: *SAINT*; program(s) used to solve structure: *SHELXS97* (Sheldrick, 2008 ▶); program(s) used to refine structure: *SHELXL97* (Sheldrick, 2008 ▶); molecular graphics: *ORTEP-3* (Farrugia, 1997 ▶) and *CAMERON* (Watkin *et al.*, 1993 ▶); software used to prepare material for publication: *SHELXL97*.

## Supplementary Material

Crystal structure: contains datablocks global, I. DOI: 10.1107/S1600536809000257/ng2532sup1.cif

Structure factors: contains datablocks I. DOI: 10.1107/S1600536809000257/ng2532Isup2.hkl

Additional supplementary materials:  crystallographic information; 3D view; checkCIF report

## Figures and Tables

**Fig. 1.**
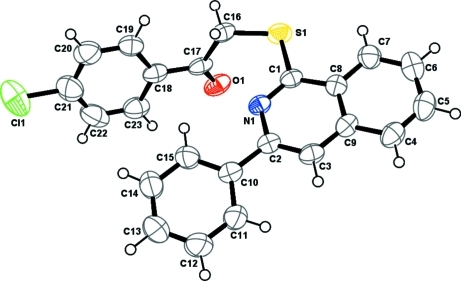
ORTEP diagram of the asymmetric unit of (I) with 50% probability displacement ellipsoids.

**Fig. 2.**
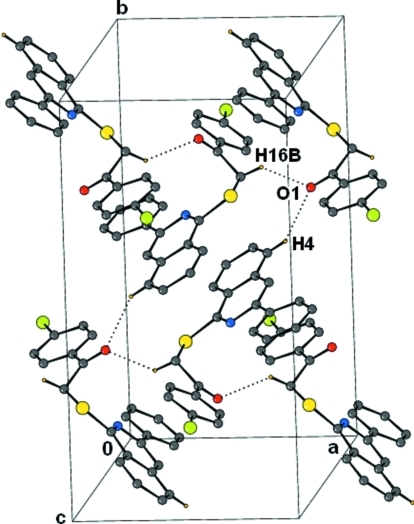
The crystal packing diagram of (I).The dotted lines indicate intermolecular C—H···O hydrogen bonds. All H atoms have been omitted for clarity.

## supplementary crystallographic information

### Comment

Organic thioethers are useful synthetic intermediates, key reagents in organic synthesis, bio-organic, medicinal, and heterocyclic chemistry (Cremlyn, 1996 and McReynolds *et al.*, 2004). They are also useful as heteroatomic functional groups in organic synthesis, for example, chiral sulphoxides can be generated by the oxidation of thioethers which are useful as auxiliaries in asymmetric syntheses (Carreno, 1995). Many syntheses have been reported for the preparation of thioethers in literature (Kondo *et al.*, 2000) whereas commonly used method is the alkylation of thiols (Mosberg *et al.*, 1985 and references there in).

The compound (I) forms the dihedral angles of 11.73 (1)° and 66.07 (1)° between mean plane of isoquinoline moiety and phenyl ring and mean plane of isoquinoline moiety and chlorophenyl ring respectively. The crystal packing is stabilized by C—H···O inter molecular Hydrogen bonds (Figure 2).

### Experimental

3-Phenylisoquinoline-1-thiol and 2-bromo-1-(4-chlorophenyl)ethanone were mixed in the ratio 1:1.05 equivalents with ethanol in a round bottom flask. Then it was heated under nitrogen atmosphere on an oil bath at 323 K. After 2 h, the products were filtered and dissolved in chloroform. Further, it was washed with water, dried and concentrated. The single-crystal for X-ray structue anlaysis was obtained from ether solution by slow evaporation.

### Refinement

All the H atoms in (I) were positioned geometrically and refined using a riding model with C—H bond lenghts of 0.93 Å and 0.97 Å for aromatic and for methylene H atoms respectively and *U*_iso_(H) = 1.2*U*_eq_(C) for all carbon bound H atoms.

### Crystal data

**Table:**

C_23_H_16_ClNOS	*F*(000) = 1616
*M**_r_* = 389.89	*D*_x_ = 1.371 Mg m^−^^3^
Orthorhombic, *P**b**c**a*	Mo *K*α radiation, λ = 0.71073 Å
Hall symbol: -P 2ac 2ab	Cell parameters from 871 reflections
*a* = 9.5874 (14) Å	θ = 1.9–25.0°
*b* = 17.888 (3) Å	µ = 0.33 mm^−^^1^
*c* = 22.025 (3) Å	*T* = 290 K
*V* = 3777.3 (10) Å^3^	Rod, colourless
*Z* = 8	0.26 × 0.20 × 0.14 mm

### Data collection

**Table:**

Bruker SMART CCD area-detector diffractometer	3332 independent reflections
Radiation source: fine-focus sealed tube	1915 reflections with *I* > 2σ(*I*)
graphite	*R*_int_ = 0.116
φ and ω scans	θ_max_ = 25.0°, θ_min_ = 1.9°
Absorption correction: multi-scan (*SADABS*; Sheldrick, 1996)	*h* = −11→11
*T*_min_ = 0.901, *T*_max_ = 0.956	*k* = −21→21
25674 measured reflections	*l* = −24→26

### Refinement

**Table:**

Refinement on *F*^2^	Primary atom site location: structure-invariant direct methods
Least-squares matrix: full	Secondary atom site location: difference Fourier map
*R*[*F*^2^ > 2σ(*F*^2^)] = 0.071	Hydrogen site location: inferred from neighbouring sites
*wR*(*F*^2^) = 0.125	H-atom parameters constrained
*S* = 1.04	*w* = 1/[σ^2^(*F*_o_^2^) + (0.0308*P*)^2^ + 0.7191*P*] where *P* = (*F*_o_^2^ + 2*F*_c_^2^)/3
3332 reflections	(Δ/σ)_max_ < 0.001
244 parameters	Δρ_max_ = 0.23 e Å^−^^3^
0 restraints	Δρ_min_ = −0.23 e Å^−^^3^

### Special details

**Table:**

Geometry. All e.s.d.'s (except the e.s.d. in the dihedral angle between two l.s. planes) are estimated using the full covariance matrix. The cell e.s.d.'s are taken into account individually in the estimation of e.s.d.'s in distances, angles and torsion angles; correlations between e.s.d.'s in cell parameters are only used when they are defined by crystal symmetry. An approximate (isotropic) treatment of cell e.s.d.'s is used for estimating e.s.d.'s involving l.s. planes.
Refinement. Refinement of *F*^2^ against ALL reflections. The weighted *R*-factor *wR* and goodness of fit *S* are based on *F*^2^, conventional *R*-factors *R* are based on *F*, with *F* set to zero for negative *F*^2^. The threshold expression of *F*^2^ > σ(*F*^2^) is used only for calculating *R*-factors(gt) *etc*. and is not relevant to the choice of reflections for refinement. *R*-factors based on *F*^2^ are statistically about twice as large as those based on *F*, and *R*- factors based on ALL data will be even larger.

### Fractional atomic coordinates and isotropic or equivalent isotropic displacement parameters (Å^2^)

**Table:**

	*x*	*y*	*z*	*U*_iso_*/*U*_eq_	
Cl1	−0.16890 (17)	0.07371 (9)	0.25163 (6)	0.1375 (6)	
S1	−0.09127 (10)	0.34062 (5)	−0.06818 (5)	0.0545 (3)	
O1	0.0295 (2)	0.19469 (13)	−0.02148 (12)	0.0588 (7)	
N1	0.0851 (3)	0.36685 (14)	0.02265 (13)	0.0406 (7)	
C1	0.0416 (3)	0.39062 (18)	−0.03051 (16)	0.0401 (9)	
C2	0.1941 (3)	0.40302 (18)	0.05067 (15)	0.0390 (9)	
C3	0.2597 (4)	0.46147 (19)	0.02355 (16)	0.0460 (9)	
H3	0.3348	0.4841	0.0430	0.055*	
C4	0.2805 (4)	0.54850 (19)	−0.06405 (19)	0.0554 (10)	
H4	0.3564	0.5724	−0.0463	0.067*	
C5	0.2337 (5)	0.5720 (2)	−0.1189 (2)	0.0644 (12)	
H5	0.2784	0.6111	−0.1387	0.077*	
C6	0.1184 (5)	0.5375 (2)	−0.14581 (18)	0.0665 (12)	
H6	0.0862	0.5543	−0.1832	0.080*	
C7	0.0532 (4)	0.47981 (19)	−0.11778 (17)	0.0570 (11)	
H7	−0.0237	0.4575	−0.1360	0.068*	
C8	0.1007 (4)	0.45349 (17)	−0.06155 (16)	0.0410 (8)	
C9	0.2160 (4)	0.48838 (17)	−0.03338 (16)	0.0428 (9)	
C10	0.2362 (4)	0.3727 (2)	0.11049 (16)	0.0439 (9)	
C11	0.3618 (4)	0.3927 (2)	0.13781 (17)	0.0656 (12)	
H11	0.4201	0.4267	0.1184	0.079*	
C12	0.4012 (5)	0.3632 (3)	0.19275 (19)	0.0801 (13)	
H12	0.4854	0.3779	0.2100	0.096*	
C13	0.3193 (5)	0.3129 (3)	0.22247 (19)	0.0778 (13)	
H13	0.3463	0.2933	0.2598	0.093*	
C14	0.1957 (5)	0.2919 (2)	0.19589 (19)	0.0748 (13)	
H14	0.1390	0.2570	0.2152	0.090*	
C15	0.1543 (4)	0.3218 (2)	0.14102 (17)	0.0594 (11)	
H15	0.0694	0.3072	0.1243	0.071*	
C16	−0.1538 (3)	0.28339 (18)	−0.00757 (16)	0.0486 (10)	
H16A	−0.1685	0.3150	0.0276	0.058*	
H16B	−0.2440	0.2634	−0.0193	0.058*	
C17	−0.0618 (4)	0.21887 (18)	0.01117 (18)	0.0454 (10)	
C18	−0.0910 (4)	0.18457 (17)	0.07171 (18)	0.0470 (9)	
C19	−0.2116 (4)	0.1996 (2)	0.10416 (18)	0.0529 (10)	
H19	−0.2767	0.2330	0.0885	0.064*	
C20	−0.2360 (4)	0.1656 (3)	0.15937 (19)	0.0693 (12)	
H20	−0.3179	0.1754	0.1806	0.083*	
C21	−0.1389 (6)	0.1174 (3)	0.18275 (19)	0.0769 (13)	
C22	−0.0177 (5)	0.1022 (2)	0.1514 (2)	0.0762 (13)	
H22	0.0480	0.0695	0.1675	0.091*	
C23	0.0055 (4)	0.1354 (2)	0.0962 (2)	0.0630 (11)	
H23	0.0869	0.1249	0.0750	0.076*	

### Atomic displacement parameters (Å^2^)

**Table:**

	*U*^11^	*U*^22^	*U*^33^	*U*^12^	*U*^13^	*U*^23^
Cl1	0.1541 (14)	0.1891 (16)	0.0694 (9)	−0.0056 (13)	−0.0200 (9)	0.0362 (10)
S1	0.0462 (6)	0.0542 (5)	0.0632 (7)	−0.0085 (5)	−0.0116 (6)	−0.0032 (5)
O1	0.0391 (15)	0.0504 (15)	0.087 (2)	0.0030 (12)	0.0112 (15)	−0.0136 (14)
N1	0.0357 (17)	0.0379 (15)	0.0481 (19)	−0.0030 (14)	0.0007 (16)	−0.0072 (14)
C1	0.032 (2)	0.038 (2)	0.050 (2)	0.0031 (16)	0.0014 (18)	−0.0074 (18)
C2	0.036 (2)	0.037 (2)	0.045 (2)	0.0020 (17)	0.0029 (17)	−0.0125 (17)
C3	0.044 (2)	0.040 (2)	0.054 (2)	−0.0063 (17)	−0.005 (2)	−0.0126 (19)
C4	0.055 (3)	0.040 (2)	0.071 (3)	−0.0047 (19)	0.010 (2)	−0.005 (2)
C5	0.074 (3)	0.043 (2)	0.076 (3)	−0.001 (2)	0.017 (3)	0.006 (2)
C6	0.084 (3)	0.058 (3)	0.058 (3)	0.005 (2)	0.003 (3)	0.011 (2)
C7	0.060 (3)	0.048 (2)	0.063 (3)	0.000 (2)	−0.004 (2)	0.003 (2)
C8	0.042 (2)	0.0353 (18)	0.046 (2)	0.0077 (17)	0.0068 (19)	−0.0053 (18)
C9	0.043 (2)	0.0303 (19)	0.055 (2)	−0.0010 (17)	0.007 (2)	−0.0096 (18)
C10	0.040 (2)	0.047 (2)	0.045 (2)	−0.0009 (18)	0.001 (2)	−0.0121 (19)
C11	0.057 (3)	0.087 (3)	0.054 (3)	−0.015 (2)	−0.002 (2)	0.004 (2)
C12	0.064 (3)	0.119 (4)	0.057 (3)	−0.018 (3)	−0.013 (3)	0.003 (3)
C13	0.082 (4)	0.101 (4)	0.050 (3)	−0.005 (3)	−0.010 (3)	0.006 (3)
C14	0.084 (3)	0.078 (3)	0.063 (3)	−0.016 (3)	−0.009 (3)	0.019 (3)
C15	0.060 (3)	0.059 (3)	0.058 (3)	−0.009 (2)	−0.011 (2)	0.005 (2)
C16	0.033 (2)	0.044 (2)	0.068 (3)	−0.0058 (17)	−0.0008 (19)	−0.0072 (19)
C17	0.031 (2)	0.0346 (19)	0.071 (3)	−0.0067 (17)	−0.005 (2)	−0.011 (2)
C18	0.039 (2)	0.0350 (18)	0.067 (3)	−0.0037 (18)	−0.005 (2)	−0.012 (2)
C19	0.047 (3)	0.053 (2)	0.058 (3)	−0.002 (2)	−0.008 (2)	−0.008 (2)
C20	0.059 (3)	0.091 (3)	0.058 (3)	−0.009 (3)	−0.006 (3)	−0.013 (3)
C21	0.084 (4)	0.090 (3)	0.057 (3)	−0.012 (3)	−0.019 (3)	−0.003 (3)
C22	0.077 (4)	0.062 (3)	0.090 (4)	0.004 (3)	−0.030 (3)	0.007 (3)
C23	0.050 (3)	0.050 (2)	0.089 (3)	0.002 (2)	−0.008 (2)	−0.001 (2)

### Geometric parameters (Å, °)

**Table:**

Cl1—C21	1.731 (4)	C11—C12	1.373 (5)
S1—C1	1.764 (3)	C11—H11	0.9300
S1—C16	1.786 (3)	C12—C13	1.362 (5)
O1—C17	1.212 (4)	C12—H12	0.9300
N1—C1	1.314 (4)	C13—C14	1.374 (5)
N1—C2	1.375 (4)	C13—H13	0.9300
C1—C8	1.433 (4)	C14—C15	1.379 (5)
C2—C3	1.359 (4)	C14—H14	0.9300
C2—C10	1.481 (4)	C15—H15	0.9300
C3—C9	1.407 (4)	C16—C17	1.510 (4)
C3—H3	0.9300	C16—H16A	0.9700
C4—C5	1.355 (5)	C16—H16B	0.9700
C4—C9	1.412 (4)	C17—C18	1.494 (5)
C4—H4	0.9300	C18—C23	1.385 (5)
C5—C6	1.398 (5)	C18—C19	1.385 (5)
C5—H5	0.9300	C19—C20	1.380 (5)
C6—C7	1.355 (5)	C19—H19	0.9300
C6—H6	0.9300	C20—C21	1.369 (6)
C7—C8	1.401 (4)	C20—H20	0.9300
C7—H7	0.9300	C21—C22	1.380 (6)
C8—C9	1.413 (4)	C22—C23	1.371 (5)
C10—C15	1.378 (4)	C22—H22	0.9300
C10—C11	1.392 (5)	C23—H23	0.9300
			
C1—S1—C16	100.46 (17)	C11—C12—H12	119.4
C1—N1—C2	119.3 (3)	C12—C13—C14	118.2 (4)
N1—C1—C8	123.6 (3)	C12—C13—H13	120.9
N1—C1—S1	119.0 (3)	C14—C13—H13	120.9
C8—C1—S1	117.3 (3)	C13—C14—C15	121.0 (4)
C3—C2—N1	121.1 (3)	C13—C14—H14	119.5
C3—C2—C10	123.1 (3)	C15—C14—H14	119.5
N1—C2—C10	115.8 (3)	C10—C15—C14	121.3 (4)
C2—C3—C9	121.2 (3)	C10—C15—H15	119.4
C2—C3—H3	119.4	C14—C15—H15	119.4
C9—C3—H3	119.4	C17—C16—S1	116.5 (2)
C5—C4—C9	121.1 (4)	C17—C16—H16A	108.2
C5—C4—H4	119.4	S1—C16—H16A	108.2
C9—C4—H4	119.4	C17—C16—H16B	108.2
C4—C5—C6	120.3 (4)	S1—C16—H16B	108.2
C4—C5—H5	119.9	H16A—C16—H16B	107.3
C6—C5—H5	119.9	O1—C17—C18	121.2 (3)
C7—C6—C5	120.5 (4)	O1—C17—C16	122.2 (3)
C7—C6—H6	119.8	C18—C17—C16	116.6 (3)
C5—C6—H6	119.8	C23—C18—C19	118.6 (4)
C6—C7—C8	120.6 (4)	C23—C18—C17	118.9 (4)
C6—C7—H7	119.7	C19—C18—C17	122.5 (3)
C8—C7—H7	119.7	C20—C19—C18	120.7 (4)
C7—C8—C9	119.6 (3)	C20—C19—H19	119.6
C7—C8—C1	123.8 (3)	C18—C19—H19	119.6
C9—C8—C1	116.5 (3)	C21—C20—C19	119.6 (4)
C3—C9—C4	123.8 (3)	C21—C20—H20	120.2
C3—C9—C8	118.2 (3)	C19—C20—H20	120.2
C4—C9—C8	118.0 (3)	C20—C21—C22	120.6 (4)
C15—C10—C11	116.9 (4)	C20—C21—Cl1	120.1 (4)
C15—C10—C2	121.4 (3)	C22—C21—Cl1	119.3 (4)
C11—C10—C2	121.8 (3)	C23—C22—C21	119.7 (4)
C12—C11—C10	121.3 (4)	C23—C22—H22	120.2
C12—C11—H11	119.3	C21—C22—H22	120.2
C10—C11—H11	119.3	C22—C23—C18	120.8 (4)
C13—C12—C11	121.3 (4)	C22—C23—H23	119.6
C13—C12—H12	119.4	C18—C23—H23	119.6
			
C2—N1—C1—C8	−0.6 (4)	C3—C2—C10—C11	−12.3 (5)
C2—N1—C1—S1	176.3 (2)	N1—C2—C10—C11	165.9 (3)
C16—S1—C1—N1	14.4 (3)	C15—C10—C11—C12	−0.4 (6)
C16—S1—C1—C8	−168.5 (2)	C2—C10—C11—C12	−178.8 (4)
C1—N1—C2—C3	−1.8 (4)	C10—C11—C12—C13	0.4 (7)
C1—N1—C2—C10	179.9 (3)	C11—C12—C13—C14	0.3 (7)
N1—C2—C3—C9	1.8 (5)	C12—C13—C14—C15	−1.0 (7)
C10—C2—C3—C9	180.0 (3)	C11—C10—C15—C14	−0.3 (6)
C9—C4—C5—C6	1.0 (6)	C2—C10—C15—C14	178.1 (3)
C4—C5—C6—C7	−0.8 (6)	C13—C14—C15—C10	1.0 (6)
C5—C6—C7—C8	−0.3 (6)	C1—S1—C16—C17	−73.9 (3)
C6—C7—C8—C9	1.2 (5)	S1—C16—C17—O1	−18.5 (4)
C6—C7—C8—C1	−176.9 (3)	S1—C16—C17—C18	163.1 (2)
N1—C1—C8—C7	−178.9 (3)	O1—C17—C18—C23	13.8 (5)
S1—C1—C8—C7	4.2 (4)	C16—C17—C18—C23	−167.7 (3)
N1—C1—C8—C9	2.9 (5)	O1—C17—C18—C19	−165.7 (3)
S1—C1—C8—C9	−174.0 (2)	C16—C17—C18—C19	12.8 (4)
C2—C3—C9—C4	−179.5 (3)	C23—C18—C19—C20	−0.8 (5)
C2—C3—C9—C8	0.7 (5)	C17—C18—C19—C20	178.7 (3)
C5—C4—C9—C3	−180.0 (3)	C18—C19—C20—C21	1.0 (6)
C5—C4—C9—C8	−0.2 (5)	C19—C20—C21—C22	−0.5 (6)
C7—C8—C9—C3	178.9 (3)	C19—C20—C21—Cl1	−179.4 (3)
C1—C8—C9—C3	−2.8 (4)	C20—C21—C22—C23	−0.2 (6)
C7—C8—C9—C4	−0.9 (4)	Cl1—C21—C22—C23	178.7 (3)
C1—C8—C9—C4	177.3 (3)	C21—C22—C23—C18	0.4 (6)
C3—C2—C10—C15	169.4 (3)	C19—C18—C23—C22	0.1 (5)
N1—C2—C10—C15	−12.4 (4)	C17—C18—C23—C22	−179.4 (3)

### Hydrogen-bond geometry (Å, °)

**Table:**

*D*—H···*A*	*D*—H	H···*A*	*D*···*A*	*D*—H···*A*
C4—H4···O1^i^	0.93	2.51	3.322 (4)	147
C16—H16B···O1^ii^	0.97	2.47	3.128 (4)	125

Symmetry codes: (i) −*x*+1/2, *y*+1/2, *z*; (ii) *x*−1/2, −*y*+1/2, −*z*.
